# A Cyber-Physical-Human System for One-to-Many UAS Operations: Cognitive Load Analysis †

**DOI:** 10.3390/s20195467

**Published:** 2020-09-23

**Authors:** Lars J. Planke, Yixiang Lim, Alessandro Gardi, Roberto Sabatini, Trevor Kistan, Neta Ezer

**Affiliations:** 1 School of Engineering, RMIT University, Bundoora, VIC 3083, Australia; lars.planke@rmit.edu.au (L.J.P.); yixiang.lim@rmit.edu.au (Y.L.); alessandro.gardi@rmit.edu.au (A.G.); 2 THALES Australia—Airspace Mobility Solutions, WTC North Wharf, Melbourne, VIC 3000, Australia; trevor.kistan@rmit.edu.au; 3 Northrop Grumman Corporation, 1550 W. Nursery Rd, Linthicum Heights, MD 21090, USA; neta.ezer@ngc.com

**Keywords:** human–machine systems, adaptive systems, one-to-many, unmanned aircraft system, UAS, neuroergonomics, electroencephalogram, EEG, eye tracking, mental workload

## Abstract

The continuing development of avionics for Unmanned Aircraft Systems (UASs) is introducing higher levels of intelligence and autonomy both in the flight vehicle and in the ground mission control, allowing new promising operational concepts to emerge. One-to-Many (OTM) UAS operations is one such concept and its implementation will require significant advances in several areas, particularly in the field of Human–Machine Interfaces and Interactions (HMI^2^). Measuring cognitive load during OTM operations, in particular Mental Workload (MWL), is desirable as it can relieve some of the negative effects of increased automation by providing the ability to dynamically optimize avionics HMI^2^ to achieve an optimal sharing of tasks between the autonomous flight vehicles and the human operator. The novel Cognitive Human Machine System (CHMS) proposed in this paper is a Cyber-Physical Human (CPH) system that exploits the recent technological developments of affordable physiological sensors. This system focuses on physiological sensing and Artificial Intelligence (AI) techniques that can support a dynamic adaptation of the HMI^2^ in response to the operators’ cognitive state (including MWL), external/environmental conditions and mission success criteria. However, significant research gaps still exist, one of which relates to a universally valid method for determining MWL that can be applied to UAS operational scenarios. As such, in this paper we present results from a study on measuring MWL on five participants in an OTM UAS wildfire detection scenario, using Electroencephalogram (EEG) and eye tracking measurements. These physiological data are compared with a subjective measure and a task index collected from mission-specific data, which serves as an objective task performance measure. The results show statistically significant differences for all measures including the subjective, performance and physiological measures performed on the various mission phases. Additionally, a good correlation is found between the two physiological measurements and the task index. Fusing the physiological data and correlating with the task index gave the highest correlation coefficient (CC = 0.726 ± 0.14) across all participants. This demonstrates how fusing different physiological measurements can provide a more accurate representation of the operators’ MWL, whilst also allowing for increased integrity and reliability of the system.

## 1. Introduction

Advancements in technologies such as Artificial Intelligence (AI), sensor networks and agent-based systems are rapidly changing the operations of Unmanned Aircraft Systems (UASs) and are introducing systems with higher levels of intelligence and autonomy [1]. Particularly, system automation is becoming increasingly complex with heterogeneous sensor networks and algorithms that incorporate increasing amount of input data and with multiple objectives. A negative effect of this complexity is the human operators’ loss of Situational Awareness (SA) and the increase in Mental Workload (MWL) in certain scenarios, where it is paradoxically meant to alleviate MWL [2]. A Cyber-Physical-Human (CPH) system is a particular class of Cyber-Physical Systems (CPS), which fundamentally addresses these issues. The implementation of a CPH system is vital as it ensures that the human maintains a central role in the operation of the system as the Human–Machine Interfaces and Interactions (HMI^2^), intelligence and autonomy advances.

The measurement of cognitive load, particularly MWL, in real-time gives CPS the ability to sense and adapt to the human operator. The proposed Cognitive Human Machine System (CHMS) is a CPH system concept that incorporates system automation support, which modulates as a function of measured cognitive state of the human operator [3,4,5]. Among other functions, the system allows dynamic adaptation of the system Automation Level (AL) and actual command/control interfaces, while maintaining desired MWL and the highest possible level of situational awareness. This new adaptive form of HMI^2^ is central to support the airworthiness certification and widespread operational deployment of One-to-Many (OTM) systems in the civil aviation context [6,7,8].

An important consideration for a CHMS is implementing a sensor network with different physiological measurements, such as those originating from electrical and metabolic brain activity, eye movement activity and cardiorespiratory activity. This is important as each physiological parameter observes different biological processes, and their corresponding sensors are thus sensitive to signal contamination originating from distinctly different disturbances. For instance, Electroencephalogram (EEG) electrodes are prone to internal and external artifacts such as eye blinks, movement, heartbeat artifacts and other electromagnetic interference [9], whereas blink rate and pupillometry are among other sensitive to ambient light stimuli [10,11]. Henceforth, in a CHMS the monitoring of multiple parameters in a sensor network ensures the integrity of the system [3]. Such a sensor network is also natively suited to exploit data fusion of the physiological measurements to increase the overall accuracy and reliability of the human operators’ estimated MWL. The disturbances mentioned above additionally mean that it is challenging to identify the true signal of interest from the noise. As such, the comparison with other MWL measures, such as subjective questionnaires and objective task performance measures, is important for cross referencing with the physiological measures, in order to verify that they are correctly and accurately measuring MWL. Moreover, having additional MWL measures is needed for potentially implementing them as labels for inference methods such as supervised Machine Learning (ML) techniques in the training/calibration phase [12].

The measurement of the physiological response and inferring cognitive states, with and without system adaptation has been demonstrated in previous studies [12,13,14,15,16,17,18]. However, there are still considerable challenges with the implementation of such methods, where some extensive reviews have identified that measures of MWL are not universally valid for all task scenarios [19,20]. A reason for this is that the physiological responses for MWL can be scenario dependent and are thus influenced by a range of individual differences and task characteristics [20].

In this paper we present a study with two physiological sensors, including an EEG and eye tracker, as well as a secondary task performance index and a subjective questionnaire as measurements of MWL in an OTM UAS wildfire detection mission. Here the participants assume the role of a UAS pilot controlling multiple Unmanned Aerial Vehicles (UAVs), where the task scenario is designed to incrementally increase in difficulty throughout a 30-min mission. This study capitalizes on existing approaches for measuring MWL and proposes a multi-sensory approach, with the data fusion of the eye tracking and EEG measures. This extends the research on the CHMS concept and demonstrates the ability to measure MWL in a complex OTM UAS task scenario. As such the contribution of this study is the relationship between the physiological and objective measures in the context of CHMS for OTM UAS operations. The contribution towards the development of a real-time measure of a human operators’ MWL will support the implementation of more adaptive and intelligent forms of automation in OTM UAS operation.

### 1.1. Background on Mental Worklaod (MWL) and MWL Measurements

Among the various forms of cognitive load, MWL is of central importance as it influences the operators’ performance and thus the system performance [21]. MWL is a complex construct and is challenging to define accurately [22], however MWL is assumed to be a reflection of the level of cognitive engagement and effort as an operator performs one or more tasks. Henceforth, a general definition of MWL is “the relationship between the function relating the mental resources demanded by a task and those resources available to be supplied by the human operator” [23]. Mental workload can thus be determined by exogenous task demands and endogenous supply of processing resources (i.e., attention and working memory). A notable distinction to make is between MWL and task load, where MWL reflects the operators’ subjective experience while undergoing particular tasks during certain environments and time constraints. However, task load is the amount of work or external duties that the operator has to perform [24]. The operators’ resulting MWL can thus be an outcome of the task demand and also endogenous factors such as experience, effort, stress and fatigue [25].

A significant human factor concern for complex, safety-critical aerospace systems is the prevention of suboptimal MWL such as mental underload and overload. Both are discriminated by referring to the source of error during operation, where the former relates to reduced alertness and lowered attention, while the latter refers to information overload, diverted attention and/or insufficient time required for information processing [2,21]. This relationship between MWL and operator performance can be modeled with the inverted U function, which indicates when an operator enters suboptimal workload that can lead to errors and accidents [26].

The present method of measuring MWL is generally done with either subjective measures, performance measures or physiological measures. Subjective measures include having the operator fill out questionnaires and self-confrontation reports such as NASA Task Load Index (NASA-TLX, [27]) and Instantaneous Self-Assessment (ISA, [28]). These measures are not in real-time and are generally collected following the completion of a task or at infrequent intervals during the experiment. Overcoming this challenge would mean interrupting the participant/operator more frequently, which would take away attention and mental resources from the primary task. Moreover, as questionnaires are self-reported the answers are prone to bias and a peak end effect [29].

Task performance measures can be further categorized into primary task performance measures and secondary task performance measures. The task performance measures generally evaluate speed or accuracy including tracking performance, reaction time or number of errors, where it can be seen as the overall effectiveness of the human machine interaction [30]. As compared to subjective questionnaires, task performance measures can be collected at much more frequent intervals. When additional tasks are added to the demand, secondary measures or dual task technique can be used. This could include the operator performing a primary task varying in cognitive demand, while having to fulfill a relatively low-demand secondary task, such as pressing a button immediately prior to hearing a tone. Here it is assumed that as cognitive capacity is increased by the primary task, there is less capacity available for the secondary task [31]. Although a more widely accepted measure for MWL, secondary task performance can be disturbing as it interferes with the primary task and may not be operationally relevant [32].

Controller inputs have also been used as a potential task-based measure of the operators’ cognitive load [33]. Among other, this measurement includes measuring the speed at which the operator responds to a task or accuracy of clicking a button. However, since reaction speed and accuracy measures are usually difficult to implement for more complex tasks, a more straightforward implementation involves the rate of control inputs, or the count of control inputs within a given time.

Lastly, physiological measures are derived from the operators’ physiology, and include measures from two anatomically distinct structures, namely the Central Nervous System (CNS) and Peripheral Nervous System (PNS) [34]. From these categories the physiological response of interest for passive control of the system are the involuntary reactive responses of the human operator [30]. These physiological measures have in recent years gained traction with the new technological developments and affordable prices and can allow for objective, unobtrusive and real-time measurement of MWL. Although there are numerous techniques for performing physiological response measures, the current notable ones include eye tracking measures, EEG, Functional Near Infrared Spectroscopy (fNIR), Electromyogram (EMG) and Electrocardiogram (ECG). In previous studies, the measurement of MWL has been demonstrated to modulate task load based on mental overload cases including the use of EEG measures in an Air Traffic Management (ATM) scenario [14]. Another study has contrarily modulated task load based on a mental underload case, where the difficulty presented to a pianist increased when an fNIR sensor detected that the presented material became too easy for the participant [18]. More commonly however, studies have mainly measured cognitive states in response to task load without dynamic task adaptation [13,15,17,35]. Nonetheless, the inference of cognitive states based on physiological data is still an active area of research, with the most promising avenue being the use of AI techniques including supervised Machine Learning (ML) to generate models of the users’ cognitive states based on labeled data [12,13,15,16,17]. For a more detailed review on the various physiological sensors, and the corresponding methods implemented for processing MWL measurements see the following reference [3].

For this study EEG and eye tracking measures were used, as such the remaining section outlines the EEG and eye tracking methods as needed for this study. When applied in clinical use, EEG frequencies bands are generally categorized into five different ranges. These include delta (δ, <4 Hz), theta (θ, 4–7 Hz), alpha (α, 8–12 Hz), beta (β, 12–30 Hz) and gamma (γ, >30 Hz) [36]. The layout of the electrode placement is standardized and follows the international 10-20 system. Previous studies have indicated that the changes in workload are observed with variations in the theta and alpha bands [35,37,38,39,40,41,42]. More specifically, with higher workload the power in the theta band has been observed to increase at the frontal and central regions [35,37,41], while in the alpha band there has been observed a decrease in power at the left and right occipital regions [41]. Additionally, previous studies have indicated that 4–6 electrodes are sufficient to achieve accurate EEG recordings of cognitive states [43].

Eye tracking features can be deduced from gaze features or pupillometry and is performed with either wearable or remote sensors. Gaze features further includes fixation, saccade, dwell, transition and scan path, while pupillometry includes eye closure, blink rate and pupil radius [44]. In regard to gaze features, the scan path can allow for more complex features to be extracted such as visual entropy [45]. The eye tracking features correlated with the cognitive state include fixation, blink rate, saccades, pupil diameter, dwell time and visual entropy [3]. Visual entropy provides a particularly useful measure, where studies have shown that visual entropy was able to discriminate between control modes and flight phases associated with different levels of MWL [46]. This measure uses the randomness of the users’ gaze patterns and once Areas of Interest (AOI) have been defined on the Human Machine Interface (HMI), visual entropy can be simply calculated from gaze data as a single, easily interpretable value.

### 1.2. Cognitive Human Machine System (CHMS) and Design Considerations

The proposed CHMS is based on an advanced CPH architecture incorporating both adaptive interfaces and automation support, which are modified dynamically as a function of the human operators’ cognitive states as well as other relevant operational/environmental observables. The counterpart of a CPH system is an Autonomous Cyber-Physical (ACP) system, which operates without the need for human intervention or control. Many of the CPS implemented today are a part of the subclass Semi-Autonomous Cyber-Physical (S-ACP) system that perform autonomous tasks in certain predefined conditions but require a human operator otherwise. However, the S-ACP systems are unable to dynamically adapt in response to external stimuli. Hence a CPH system addresses this as the interaction between the dynamics of the system and the cyber elements of its operation can be influenced by the human operator, and the interaction between these three elements are continuously modulated to meet specific objectives.

**Figure 1:**
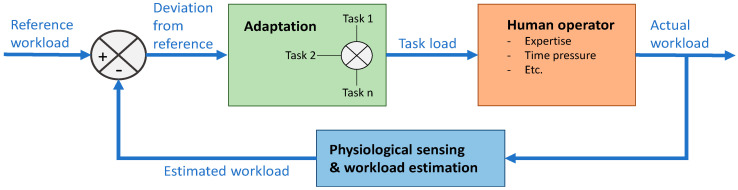
Negative feedback loop for passive control of a system.

A key feature of the CHMS, initially described in [4,5], is the real-time physiological sensing of the human operator to infer cognitive states that drive system adaptation. In its fundamental form, the CHMS framework can be depicted as a negative feedback loop as seen in Figure 1 below. Here, MWL is used as the reference for modulating the automation support and interface, where the resulting MWL for the human operator is a function of the task load (i.e., the number of tasks and/or task complexity) and the operators’ endogenous factors (i.e., expertise, time pressure, etc.). Hence, when the workload is measured to increase or decrease beyond the specified thresholds, the adaptation module is activated to modulate the operators’ task load, which can be done by changing the automation level, task scheduling and/or changing the interface. The operation of CHMS is expected to provide benefits for several aerospace areas apart from OTM UAS operations including ATM [47], Urban Traffic Management (UTM) [48] and Single Pilot Operation (SPO) [5,7]. The operation of the CHMS in all these applications will support the systems to operate at higher levels of autonomy while ensuring that the human operator maintains a central role of the system and the degree of trust with the system is maintained.

**Figure 2:**
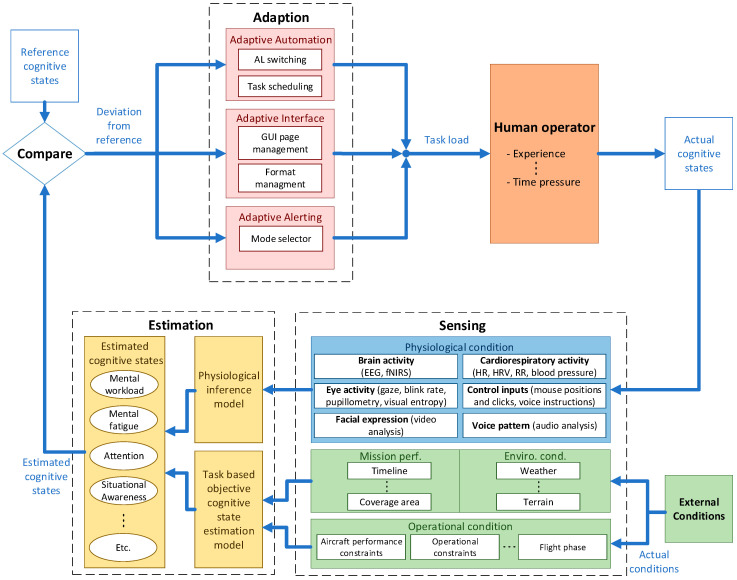
Cognitive Human Machine System (CHMS) framework.

The CHMS has parallels to a passive Brain Computer Interface (pBCI) [49], however CHMS further expands on pBCI by implementing other physiological parameters apart from brain signal processing and additionally incorporates external environmental/operational factors for estimating the cognitive states. The more detailed CHMS concept is depicted in Figure 2 and requires the adoption of three fundamental modules: sensing, estimation and adaptation. The sensing module includes two sensor networks including the sensors for measuring physiological and external conditions. The physiological sensors include various advanced wearable and remote sensors, such as the EEG and eye tracker. The other network includes for example sensors for measuring weather and measurements about the flight phase. The collected data is then passed to the estimation module, where the data from the networks are passed to respective inference models. This is then combined to make a final estimation of the different levels of the cognitive states. The estimated cognitive states are then compared with the reference cognitive states, and the deviation from these predefined references is what drives the adaptation module, which includes changing the AL, task scheduling, the interface and/or the alerting mode. These alterations thus modify what information and tasks are presented to the human operator, which again alters the cognitive states of the human operator, and the cycle then continues.

Before full implementation of a CHMS in future operational use, an initial training/calibration phase would need to be performed to calibrate the estimation module by generating and validating a cognitive state model of the human operator. Such a calibration phase will define the baseline and thresholds of the cognitive states, which will serve as the reference cognitive state conditions for comparison with the operationally collected and estimated data. The inference method adopted for the CHMS estimation module can include various AI methods, where supervised ML models are among the most promising approaches [12]. With such a method however, the calibration phase should be conducted using additional objective measures, such as secondary task performance, task complexity (determined analytically prior to the experiment) and/or controller inputs, which will serve as data labels for model training/calibration.

As mentioned above, the various physiological sensors and their biological processes are prone to distinctly different disturbances. Although multiple sensors are needed to improve reliability, some challenges arise with this including different measurement performance (e.g., accuracy, resolution, etc.) and sampling frequencies of each sensor. As such a sensor network optimisation scheme is key when designing a reliable CHMS [3]. The adoption of sensor networks is both a natural and necessary evolution to effectively exchange, synchronise and process measurement data within a customisable operational network architecture. In addition, a sensor network is natively suited to exploit data fusion of the physiological measurements to increase the overall inference accuracy and reliability of the estimation module.

The remaining sections of this paper outlines the materials and method, results, discussion and conclusion. In Section 2, materials and methods for this study are described and include details on the task scenario as well as the methods for the post processing analysis implemented. The following section are the results, which comprises of two parts. The first part presents a statistical comparison between the mission phases (Phase 1, 2 and 3) for all the MWL measures, including subjective, performance and physiological measures. The second part of the results section provides a correlation analysis of the continuous physiological measures (EEG and eye tracking) and the continuous performance measures. Furthermore, a method for fusing the physiological measures is implemented and analyzed. Lastly, the results are discussed, before a conclusion is drawn in Section 5.

## 2. One-to-Many UAS Test Case

### 2.1. Participants

There were five participants that took part in the experiment comprising of four males and one female. The participants were aerospace students at Royal Melbourne Institute of Technology (RMIT) University and were selected based on their prior experience in aviation and aerospace engineering. None of the participants had prior experience with this OTM scenario, and as such two different familiarization sessions were conducted lasting around an hour each. All participants volunteered for the experiment and were not paid. Informed verbal consent was given prior to the experiment. The corresponding ethics approval code for this research is ASEHAPP 72-16.

### 2.2. Experimental Procedure

The experimental procedure consisted of a briefing, sensor fitting and a rest period, followed by the mission. After the mission was completed, there was a second rest period before a final debrief. The whole procedure took approximately one hour. The refresher briefing was conducted to ensure that participants were familiar with the scenario and the interface. Following that, participants were fitted with the EEG device and the EEG electrodes impedances were checked to ensure they were within acceptable levels, this was then followed by a calibration of the desk-mounted eye tracker. Once both sensors were set-up, physiological data recording started, and data was logged for 5-min while the participant rested. After the resting phase the OTM UAS wildfire scenario commenced, which consisted of three back-to-back 10-min phases designed to provide increasing levels of difficulty. At the end of the scenario, physiological data was logged for another 5-min during a post-mission resting phase. Subsequently, participants provided subjective ratings for their workload and situational awareness in each of the three phases.

### 2.3. Mission Concept

For this scenario the test subjects assume the role of a UAS ground operator tasked with coordinating the actions of multiple UAVs in a wildfire surveillance mission. The primary objective of the mission is to find and localize any wildfires within the Area of Responsibility (AOR). The secondary objectives are to firstly maximize the search area coverage, and secondly to ensure that the UAV fuel levels, as well as navigation and communication (comm) performance are within a serviceable range. Further details about the mission objectives are provided in Table 1.

The sensor payload of the UAV comprises of an active sensor (lidar) and a passive sensor (Infrared (IR) camera). UAVs can be equipped with either one of the two sensors or both sensors. The lidar provides an excellent range but a narrow field of view. To operate the lidar, it must be fired towards a ground receiver to measure the CO_2_ concentration of the surrounding atmosphere (i.e., the mean column concentration of CO_2_), and areas with excessive CO_2_ concentration are likely to contain wildfires. There are a limited number of ground receivers within the AOR, which constrains the search area of the lidar. On the other hand, the infrared camera possesses a smaller range but has a larger field of view. Unlike the lidar, the camera does not require the use of a ground receiver and can be used anywhere within the AOR.

**Figure 3:**
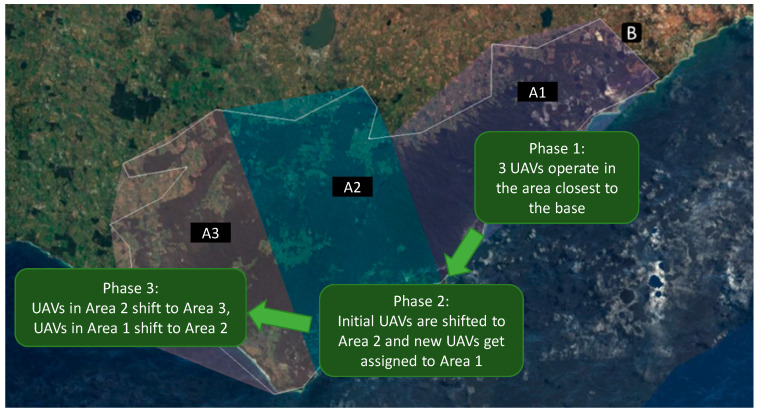
Mission concept illustrating the Unmanned Aerial Vehicles (UAVs) bounding strategy.

The AOR is divided into smaller regions called Team Areas, which can then be assigned to UAV Teams. The division of the AOR into smaller regions allows UAVs to bound from area to area, initially conducting the search in the area closest to the base before searching further out. The concept for this is illustrated in Figure 3 with the AOR denoted in white borders while the Team Areas are depicted as convex polygons of different colors. In Phase 1 of the scenario, 3 UAVs are made available to the human operator to search the area closest to the base (Team Area 1). After the Area has been searched, or when the mission transits to Phase 2 (whichever occurs first), the operator will direct the initial UAVs, originally in Team 1, to move to Area 2 in order to allow the new UAVs to take over the coverage of Area 1. After Area 2 has been searched, the human operator repeats the same strategy with Area 3, moving the UAVs originally in Area 2 into Area 3 and the UAVs originally in Area 1 into Area 2. UAVs assigned to search an area should be assigned to the team associated with that area (i.e., Team 1 for Area 1, Team 2 for Area 2, etc.), as the team structure allows operators to exploit some built-in automation support such as search area designation, path planning and platform allocation. For further detail on the concept of operations and task analysis see the following references [50,51].

Depending on how the scenario evolves the MWL profile during this mission can be different between one participant to another. Nonetheless, although a simpler scenario can generate a more repeatable MWL profile, a more realistic scenario was used to evaluate the feasibility of the OTM concept and to allow for known physiological measures to be tested on a realistic application. Repeatability was maximized by carefully controlling independent variables such as the number of UAVs being controlled and the geographic extent of the AOR over each phase of the mission.

#### Secondary Task Index

A task index was used to provide an additional objective and continuous measure of MWL during the scenario. The main purpose of the task index was to assess the secondary task performance of the participant by providing a weighted count of the number of pending secondary tasks (i.e., system maintenance tasks). The number of pending tasks was calculated from the UAV flight logs as detailed in Table 2 below. Each UAV can thus have up to 6 points at any given time indicating a high level of unsatisfactory secondary task performance.

### 2.4. Equipment and Measurement Methods

#### 2.4.1. Eye Tracker Equipment and Data Processing

The eye tracking data was collected using the Gazepoint GP3, which is a remote sensor positioned at the base of the monitor about 65 cm away from the participant. The raw eye tracking data comprises of the x and y coordinates of the gaze point and the blink rate. The system is setup to take the average x and y coordinates from the left and right pupil. If one pupil is not detected the system takes the x and y coordinates of the remaining pupil. If both are not available an invalid data point is recorded which will not be included in the data analysis. To allow for real-time processing of the scenario parameters and the processing of the eye tracking measurements, all eye-tracking data was routed to a central server. Besides eye tracking data, the server also collects and processes the flight logs of each UAV, each including the position, attitude, task type, autopilot mode, automation mode and performance of the different subsystems. During the scenario, the raw eye tracking data was processed by the server to derive other real-time metrics, including: dwell time on UAVs and UAV teams, attention on UAVs and UAV teams, along with UAV and team visual entropies, calculated from separate transition matrices of UAVs and UAV teams. However, visual entropy for UAVs gave the best indication of workload and was thus solely used for further analysis.

The visual entropy (H) is determined from gaze transitions between different Regions of Interest (ROIs), which are typically represented in a matrix. The cells represent the number (or probability) of transitions between two interfaces. The visual entropy measures the randomness of the scanning patterns, and is given by [45]:

(1) $$H=\;-\overset{n}{\underset{i=1}{\sum }}p\left(X_{i}\right)\overset{m}{\underset{j=1}{\sum }}p\left(Y_{ij}|X_{i}\right)\;\log_{2}p\left(Y_{ij}|X_{i}\right)$$

where n and m are the rows and columns of the transition matrix respectively, $p\left(Y_{ij}|X_{i}\right)$ is the probability of fixation of the present state (i.e., fixation at region $Y_{ij}$ given previous fixation at region $X_{i}$) and $p\left(X_{i}\right)$ is the probability of fixation of the prior state (i.e., probability of the previous fixation). A high value of H implies high randomness in the scan path while a low value of H implies an orderly scan pattern, therefore higher values of H indicate periods of higher workload where the operator is unable to maintain a regular scan pattern.

#### 2.4.2. EEG Equipment and Data Processing

For performing the EEG recordings during the experiment, the actiCAP Xpress, from Brain Products GmbH was used. The EEG device utilizes low impedance gold-plated electrodes, which are meant to optimize the connectivity, thus reducing the need for electrode gel. However, from observation it was found that electrode gel was needed to obtain a clear signal. Moreover, the cap is combined with the V-Amp amplifier, and the software Brain Vision Recorder, which is used for visualizing and storing the raw EEG data. The layout of the cap follows the international 10–20 system, where 16 data electrodes were collecting data at the locations F4, Fz, F3, FC1, FC2, C3, C4, CP1, CP2, T7, T8, P3, Pz, P4, O1 and O2. The active reference electrode and passive ground electrode are placed on the earlobes of the participant. While being fitted with the EEG the minimum impedance accepted was below 5 kΩ. To achieve this the unsatisfactory electrode was either jiggled or alcohol and/or gel was applied to the area. The resulting EEG index is as described in the equation below, were it is calculated at 5 s intervals:

(2) $$EEG\;index=\frac{\theta_{F4+C4}}{\alpha_{O1+O2}}=\;\frac{\int_{4hz}^{7hz}f\left(\lambda \right)d\lambda }{\int_{8hz}^{12hz}f\left(\lambda \right)d\lambda }$$

here $\theta_{F4+C4}$ refers to the average theta power for electrode positions F4 and C4, while $\alpha_{O1+O2}$ average alpha power for positions O1 and O2. This was achieved by initially processing the individual channels with a bandpass filter between 0.5 and 30 Hz. A five second sample window was then applied for each channel to obtain fixed-length signal samples, which are then preprocessed by applying linear detrending. The Power Spectral Density (PSD) of the filtered sample window is then obtained and then the respective bands are integrated to determine the band power. Once all channels have been processed, the band powers of the respective channels are averaged and then divided to derive the EEG index. After the EEG index was calculated for all the 5 s intervals additional smoothing was performed prior to the data analysis. This was done using a lowpass filter and highlighted the predominant trends in the data.

For the EEG data processing, additional data rejection criteria was included where data identified as outliers were removed. Here the isoutlier function in MATBLAB was used, which returned true for all elements that were more than 3 standard deviation from the mean. The function was performed following the calculation of the EEG index. Among the data for all the participants there were identified outliers for one participant. Here 5 outliers were detected, which were then replaced with mean values.

#### 2.4.3. Controller Input Processing

During the scenario the subject controlled and navigated the application by clicking on the screen with the left and right mouse buttons. The mouse clicks were logged by the central server and total number of controller inputs (number of left and right clicks) was counted during 2-min intervals. Additional processing was obtained to discriminate between command inputs and panning/zooming inputs, however these results are not presented here.

### 2.5. Data Analysis

For data analysis the multiple one-way Analyses of Variance (ANOVA) and Pearson correlation coefficient was carried out on the processed data. A 5% significance level was used for all the statistical tests.

#### 2.5.1. ANOVA Analysis

Multiple one-way Analyses of Variance were carried out to determine the statistical significance of the dependent measures in the different phases of the test scenario. The dependent measures comprised of a subjective questionnaire, physiological measures and performance measures. Physiological features and task performance measures were post-processed to obtain the normalized mean values for each participant in each phase of the test, comprising of five phases: Pre-rest, Phase 1, Phase 2, Phase 3 and Post-rest. Values were normalized using the data collected from all five phases of a participants’ dataset and were centered to have a mean of 0 and scaled to provide a standard deviation of 1. Additionally, the Tukey’s test was further implemented to identify what groups are significantly different from one another.

#### 2.5.2. Correlation between Features

To investigate the linear relationship between the features, the Pearson Correlation Coefficient (CC) was calculated from all combinations of the different measurements. Equation (3) outlines the correlation coefficient:

(3) $$CC=\frac{n\left(\sum xy\right)-\left(\sum x\right)\left(\sum y\right)}{\sqrt{\left[n\sum x^{2}-{\left(\sum x\right)}^{2}\right]}\left[n\sum y-{\left(\sum y\right)}^{2}\right]}$$

here *n* is the number of data points while *x* and *y* are the two respective features being analyzed. For each participant, pairwise correlations between six features were calculated. These six features include the EEG index, visual entropy, task index, control inputs, fused physiological measure and fused objective measure. The fused physiological measure was made up of a weighted sum of the visual entropy and EEG index while the fused objective measure was made up of a weighted sum of the task index and control inputs. Three different sets of weights were explored including 50/50, 70/30 and 30/70. As each participant had an individual correlation coefficient value for each feature-pair, a single value was obtained by determining the mean and standard deviation of that feature-pair across all participants.

## 3. Results

### 3.1. ANOVA Analysis

The ANOVA analysis was conducted to determine if there were significant differences in both dependent measures across the different mission phases, giving an insight into the experimental design of the scenario and if the results are in fact suitable for further analysis and implementation. The dependent measures included in the ANOVA analysis comprised of subjective ratings, physiological features and performance measures. The subjective ratings included the mental workload rating and the situational awareness rating for each mission phase. The performance measures included the average task index value and controller input count across each phase, while the physiological measures included the average value of EEG index and visual entropy in each phase. The EEG index measurement was performed during the pre- and post-mission resting stages and was thus analyzed for all five phases as well as just the three mission phases. The results of the ANOVA analysis are summarized in Table 3 below.

#### 3.1.1. Subjective Rating

**Figure 4:**
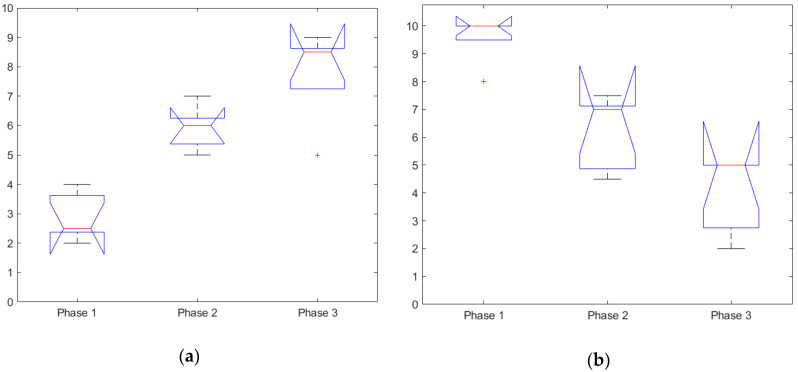
Subjective ratings: (**a**) subjective workload of all participants, grouped by the scenario phase and (**b**) subjective situational awareness of all participants, grouped by the scenario phase.

The ANOVA test for the subjective workload rating showed subjective workload to be significant, F(2,12) = 24.09, *p* = 6.28 × 10^−5^, see Table 3 and Figure 4a. Further post hoc comparison using the Tukey HSD test indicated that all 3 groups were significantly different from one another, Phase 1 (M = 2.90, SD = 0.503), Phase 2 (M = 5.9, SD = 0.503) and Phase 3 (M = 7.80, SD = 0.503).

Performing the ANOVA test for the subjective situational awareness rating demonstrated that the SA rating was significant F(2,12) = 25.82, *p* = 4.49 × 10^−5^, see Table 3 and Figure 4b. Post hoc comparison using the Tukey HSD test showed that all 3 groups were significantly different from one another, Phase 1 (M = 9.6, SD = 0.555), Phase 2 (M = 6.2, SD = 0.555) and Phase 3 (M = 4, SD = 0.555).

These results indicate that the experimental design for the mission scenario was successful in increasing the task load and mission complexity across the three mission phases, as indicated by an increasing MWL and decreasing SA. Although the subjective measures are prone to bias and the measures are infrequent, these results could serve as an additional reference for the physiological measure and are useful for comparison between the different ANOVA analyses.

#### 3.1.2. Task Index and Controller Input

**Figure 5:**
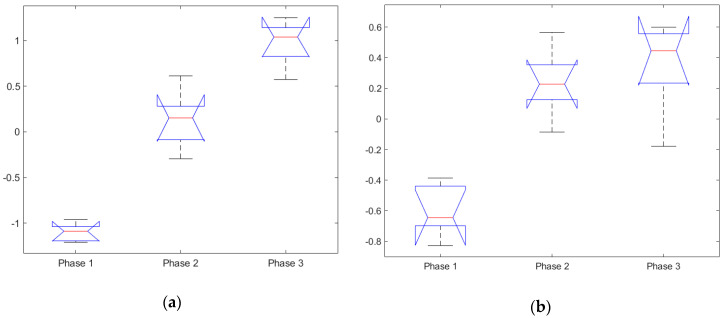
(**a**) Mean task index of all participants (normalized), grouped by the scenario phase and (**b**) mean controller input count of all participants (normalized), grouped by the scenario phase.

The ANOVA test performed on the task index showed it to be significant, with F(2,12) = 88.47, *p* = 6.56 × 10^−6^, see Table 3 and Figure 5a. The Tukey HSD test indicated that all 3 groups were significantly different from one another, Phase 1 (M = − 1.102, SD = 0.111), Phase 2 (M = 0.125, SD = 0.111) and Phase 3 (M = 0.977, SD = 0.111). These results were the strongest among all the various measures, and thus indicate the task index could serve as a proxy to measuring MWL. This result also supported the comparison with the subjective MWL measures that similarly increases between the phases.

ANOVA showed the effect of controller input to be significant, with F(2,12) = 22.1, *p* = 9.47 × 10^−5^, see Table 3 and Figure 5b. Post hoc comparison using the TUKEY HSD test showed that Phase 1 (M = − 0.594, SD = 0.110) was significantly different from both Phases 2 and 3, while controller input in Phases 2 and 3 were not significantly different from each other. The lack of significant difference in Phases 2 and 3 implies that the control input count might not be a suitable proxy for MWL at medium to high workload levels since it tended to saturate at these stages, leading to decreased sensitivity.

#### 3.1.3. Physiological Measures

**Figure 6:**
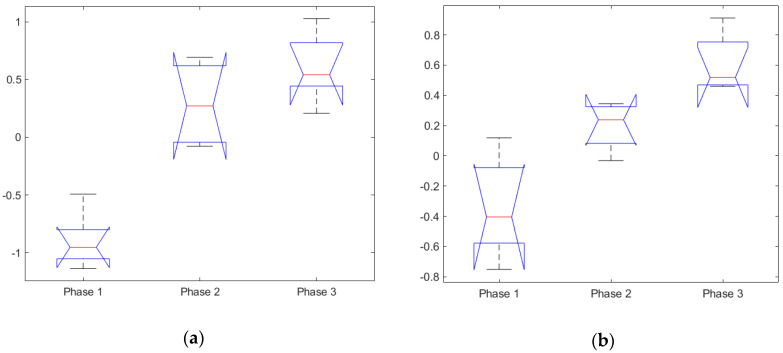
Physiological measures: (**a**) mean visual entropy of all participants (normalized), grouped by the scenario phase and (**b**) mean Electroencephalogram (EEG) index of all participants (normalized), grouped by the scenario phase.

ANOVA test showed visual entropy to be significant, F(2,12) = 34.54, *p* = 1.05e-05, see Table 3 and Figure 6a. Further post hoc comparison using the Tukey HSD test showed that Phase 1 (M = − 0.903, SD = 0.136) was significantly different from Phase 2 and 3, while the visual entropy in Phase 2 and 3 was not significantly different from each other. Although the means were increasing in line with subjective MWL measure and the task index measure, these were not statistically significant. One reason for the lack of statistical difference is that the visual entropy measure loses sensitivity between the medium and high workload.

For the EEG index the ANOVA analysis was performed on both the mission Phases 1, 2 and 3, as well as the performing the test on all five phases, where Pre- and Post-rest Phases were included. For the mission Phases 1–3 only, the ANOVA showed that the EEG index was significant, F(2,12) = 19.57, *p* = 0.0002, see Table 3 and Figure 6b. Further post hoc comparison using the Tukey HSD test showed that all three groups were significantly different from one another, Phase 1 (M = − 0.340, SD = 0.108), Phase 2 (M = 0.198, SD = 0.108) and Phase 3 (M = 0.612, SD = 0.108). For this analysis these results were the best for the physiological measures as all three groups were significantly different from one another. Moreover, this is comparable with the analysis on the subjective MWL measure and the task index measure.

**Figure 7:**
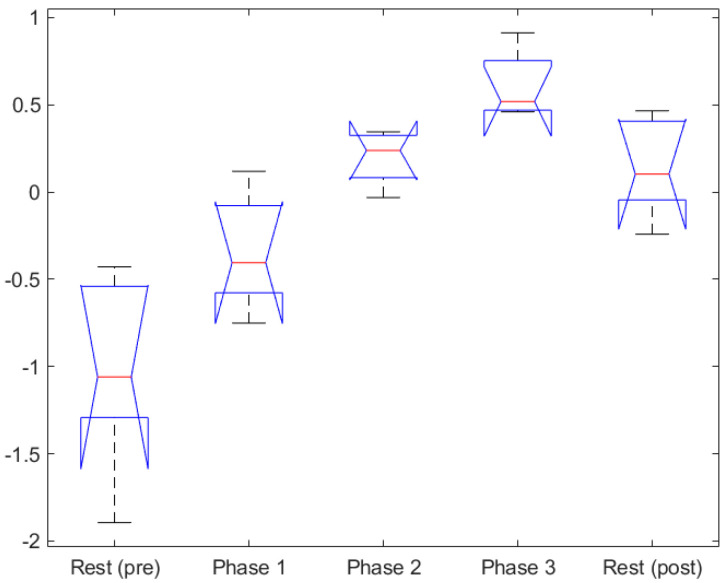
Mean EEG index of all participants (normalized), grouped by scenario Phase 1, 2 and 3 as well as Pre- and Post-resting.

For the ANOVA test performed on the full experiment length showed that the EEG index was significant, F(4,20) = 16.44, *p* = 4.11 × 10^−6^, see Table 3 and Figure 7. Furthermore, the Tukey HSD test showed that the Pre-rest Phase (M = − 1.01, SD = 0.153) was significantly different from the four other groups. Phase 1 (M = − 0.34, SD = 0.153) was significantly different from Phase 3 and the Pre-rest Phase, while Phase 2 (M = 0.19, SD = 0.153) and Post-rest Phase (M = 0.15, SD = 0.153) were only significantly different to the Pre-rest Phase. Lastly, Phase 3 (M = 0.61, SD = 0.153) was significantly different to Phase 1 and the Pre-rest Phase. For these results the expected response would be that the Pre- and Post-resting Phases are similar (or not statistically different), while Phases 1, 2 and 3 are different. Nonetheless, with the exception of the Post-resting Phase, the means of the phases were statistically different. This can be seen when performing the ANOVA and TUKEY test, while excluding the Post-rest Phase. This could be a consequence of the notion that the protocol for post resting was not well enough enforced. This analysis indicates that EEG index could further discriminate between a Pre-resting Phase and the mission Phases 1–3.

The ANOVA results show that controller input and visual entropy analysis can both discriminate Phase 1 from Phases 2 and 3 but failed to be statistically significant between Phases 2 and Phase 3. On the other hand, the subjective MWL measure, task index measure and the EEG index measure show greater statistical significance. These three measures show a similar effect of an increasing mean across the three mission phases, strongly corroborating the three different MWL measures (subjective, task-based and physiological) and showing that the experimental results were in-line with expectations.

### 3.2. Correlation Between Features

**Figure 8:**
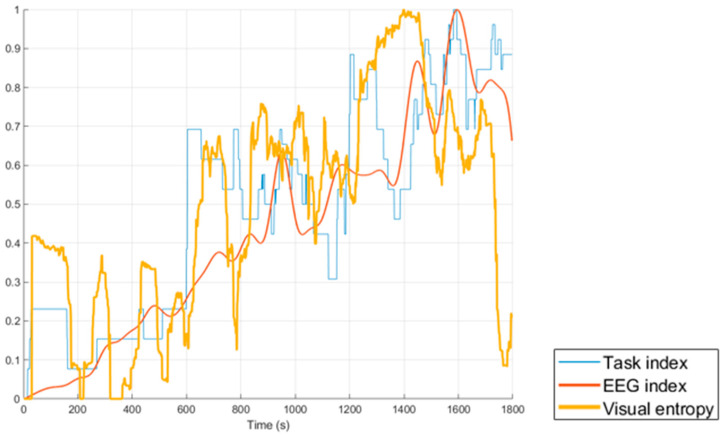
Comparison for one participant between the task index (blue) and the two physiological measures visual entropy (yellow) and EEG index (red).

Further results include the correlation between the time series of the different features. Figure 8 plots the results for one participant and shows the comparison between the task index (blue) and the two physiological measures visual entropy (yellow) and EEG index (red). The x axis is time in seconds, while the values are normalized between 0 and 1 for visual comparison and statistical analysis.

Table 4 summarizes the correlation coefficient values of the most notable features for each participant. These include the correlation between the task index and (1) the EEG index, (2) visual entropy and (3) the fused weighted sum of the two physiological measurements (weighted 50% each). Additionally, the correlation between the two physiological measurements, EEG index and visual entropy, were compared for all participants. As fulfilled by the data rejection criteria a section of the eye tracking data for participant 2 was excluded from the analysis and excluding this invalid data did improve the pairwise correlation.

Table 5 presents the pairwise correlation coefficient values in the matrix form. The values were combined across all participants by taking the mean and standard deviation. The results indicate that there was no correlation between the control input and other features. However, the mean for all the participants shows that the correlation between the task index and fused physiological feature (a 50–50 weighted sum of the EEG index and visual entropy) was highest at CC = 0.726 ± 0.14. The second highest correlation was between the task index and the visual entropy with a CC = 0.648 ± 0.19. The mean correlation between EEG index and the task index gave CC = 0.628 ± 0.17, while the mean correlation between the EEG index and visual entropy was CC = 0.561 ± 0.11.

Further analysis to explore the effects of differently weighted ratios showed that when weighting the visual entropy measurement 30% and the EEG index 70% the correlation with the task index and the fused sensors gave CC = 0.710 ± 0.16. When weighting the visual entropy measurement 70% and the EEG index 30% the correlation coefficient was CC = 0.710 ± 0.14.

The correlation of the time series for the different features show that no or poor correlation between the control input and other features was found. However, a good correlation was found between the task index and the fused sensor measurements as well as between the task index and the EEG index/Visual entropy. In addition, the correlation between the two physiological measurements was shown to be good. Weighting the physiological features 70/30 or 30/70 did not have much effect on the result as they remained strong.

## 4. Discussion

This study provided insight into the relationship between physiological and objective measures in a OTM UAS operation. However, in addition to this a number of useful insights were provided into the role of automation support in a multi-UAS context. The ground operators’ main responsibilities included routine monitoring of UAV system health, analysing sensor data and strategically ensuring that resources were appropriately allocated within the AOR when planning UAV sorties or retasking individual UAVs. While the scenario was relatively manageable when participants were controlling three UAVs, they found it more challenging in the later phases when controlling more than six UAVs. Mission complexity was generally observed to scale exponentially with the number of UAVs, primarily due to the exponentially increasing number of interactions between different platforms in addition to the linearly increasing number of system monitoring and sensor analysis tasks. In this context, the automation support provided was aimed to reduce scenario complexity by taking over some of the tasks associated with managing the interactions between platforms. This was achieved by the UAV Team concept where UAVs were grouped into teams, allowing participants to stay ‘on-the-loop’ by managing the behaviour of UAV teams instead of remaining ‘in-the-loop’ by individually micromanaging each UAV. This behaviour was evident during the experiment, as participants tended to maintain better situational awareness when managing UAVs in teams. It was also observed that participants preferred to micromanage a small number of UAVs in the initial phase of the scenario but switched to team management in the latter two phases. Participants who did not make the switch to team management provided feedback that they did not trust the automation support as it was not sufficiently transparent or reliable. Another important observation was that even under team management mode, participants were still required to allocate significant attentional resources to micromanaging individual UAVs at specific instances in the mission (e.g., when troubleshooting system health, retasking the UAV or manually controlling the sensor to localize a fire), effectively transitioning from ‘on-the-loop’ command to ‘in-the-loop’ control. It was however observed that participants sometimes failed to assume direct control of UAVs when appropriate (e.g., when user input was required to resolve an issue with the system health), either because they were focused on another task, were overwhelmed by the amount of information/pending tasks that they overlooked the particular UAV, or because they assumed that automation support was capable of resolving the issue. As such the development towards adaptive interfaces are expected to support better transitions between ‘on-the-loop’ and ‘in-the-loop’ command, as it is able to infer the users’ workload, intention and allocation of attentional resources and subsequently vary the amount of on-screen information to ensure smoother transitions.

As for the statistical analysis in this study, the ANOVA and correlation coefficient were both used to highlight two different factors. The ANOVA test was performed in the initial analysis and served to determine the validity of the experimental design as well as to get an idea of the average values of each measure across the different scenario phases. Following the ANOVA, a more detailed comparison of the time series data was carried out by evaluating the pairwise correlation coefficients between the various performance and physiological measures.

The results for the ANOVA analysis showed that all the measures were statistically significant, however a further Tukey test demonstrated that measures with all three scenario phases statistically differentiating from one another included the subjective responses for MWL and SA, as well as the task index and EEG index. As for the control input count, the results indicated that implementing this as an objective measure of MWL may not be a viable option. However, it can be noted that as the scenario is designed to push MWL to the limit it could be observed that the control input count saturated with high load and lost sensitivity between Phases 2 and 3. This means that further work remains to determine whether there are correlations between physiological measures and control input count to a system. As for the visual entropy, although not statistically different for all phases, the data was invalid for one participant during an extended period of the experiment. This occurred when the participant moved out of range of the camera, causing the eye tracker to lose track of the participants’ pupil. Further investigating the CC results of that eye tracking measure showed that when excluding the section where the data was lost (at the start of Phase 3) and then correlating with the other measures the correlation improved. This highlights the importance of having at least two physiological sensors implemented in a CHMS system, since physiological observables can be particularly affected by noise, motion artifacts or susceptible to interference due to participant movement. Multiple sensors can additionally increase the consistency of measurements and reliability of the system. Performing the ANOVA test and corresponding Tukey test demonstrated firstly that the subjective workload and situational awareness ratings, which serves as the best approximation to the ground truth, was consistent with the results for the task index and the EEG index during Phases 1, 2 and 3. Although the task index and EEG index values were averaged across each of the three 10-min phases, it provided an initial assessment to determine what measures are suitable for further analysis.

The correlation between the time series measures using the CC demonstrated how the various performance and physiological measures compared in a complex OTM UAS task scenario. While subjective ratings are currently the best approximation to ground truth, these can only be taken after extended periods of time (e.g., at the end of each phase). However, the actual workload and situational awareness of the participant can fluctuate significantly throughout each of the 10-min phases. For example, sudden spikes in the task index were observed at the start of each phase for most participants since this was a period where new UAVs were released. The task index was thereafter observed to decrease or stabilize and only peak when the participant experienced increased load such as when localizing fires or retasking UAVs. These fluctuations in mission difficulty within each phase cannot be captured by subjective questionnaires. When comparing the task index with the EEG index and visual entropy, a relatively high correlation was expected, as they are supposed to fundamentally measure the similar variation in MWL. The difference being that the EEG index and visual entropy are physiological measures, while the task index is a task-based performance measure. Looking at Figure 8, the graphical comparison illustrated that visual entropy correlated with the task index in certain regions where the EEG index does not, and vice versa. Showing that the two physiological measures, although both gradually increasing, respond differently to the task demand of the scenario in short timeframes. The weighted sum of the two physiological measures (a 50–50 weighted sum of the EEG index and visual entropy) demonstrated a higher correlation with the task index (CC = 0.726 ± 0.14) than each individual physiological measure. This further demonstrates the importance of having more physiological sensor measurements and fusing methods when performing measurements and estimations on MWL in a fully operational CHMS system. Different weighted sums were also explored including weighting the visual entropy measurement and the EEG index 30–70% and 70–30% respectively. However, changing the weights did not show much effect. This can potentially be improved with an optimal weighting strategy that is unique for each individual subject.

The concluding results thus show that a moderate level of correlation was found across all participants between the task index and EEG index CC = 0.628 ± 0.19, as well as task index and visual entropy CC = 0.648 ± 0.17. Additionally, a fusing method demonstrated that fusing the physiological measures produced an improved and a high-level correlation CC = 0.726 ± 0.14. These results indicate that the physiological response of MWL for EEG and eye tracking are consistent with previous studies. This includes the EEGs observation of fluctuation in theta power in frontal and central regions and alpha power fluctuation in parential and occipital regions during increased mental task demand [35,37,41]. Similarly, visual entropy has been shown to correlate with higher mental demand [45,46]. Nonetheless, the measures of the physiological response of MWL were conducted on a new type of mission scenario. The mission specific task index was also introduced to provide an additional baseline for comparing the EEG and visual entropy measures. Henceforth, the significance of this study is the verification of established physiological response measures of MWL, including EEG and eye tracking, as well as the relationship between the physiological and objective measures in a complex OTM UAS wildfire detection scenario. The verification of a multi-sensor fusion method additionally demonstrates that the approach can improve the reliability of cognitive state measurements. Moreover, the demonstration of a highly correlated objective measure can provide useful for potential use as labels for the physiological data when implementing AI techniques such as supervised ML models.

Future research includes exploring different data fusion techniques including further testing an optimal weighting strategy that is calibrated for each individual subject. Additional future research includes testing the objective performance measures (i.e., a secondary task performance measure) as labels for AI techniques such as supervised ML models.

## 5. Conclusions

Recent developments in avionics hardware and software for Unmanned Aircraft Systems (UASs) are introducing higher levels of intelligence and autonomy, which in turn facilitate the introduction of new advanced mission concepts such as One-to-Many (OTM) UAS operations. However, the effective implementation of OTM operations in current and likely future UAS missions will have to rely on substantial advances in the field of Human–Machine Interfaces and Interactions (HMI2). Particularly as negative effects arise with the increasingly more complex system automation, such as the human operators’ loss of situational awareness and the increase/decrease in Mental Workload (MWL). The Cognitive Human Machine System (CHMS) systems presented in this paper implements an innovative Cyber-Physical-Human (CPH) system architecture that incorporates real-time adaptation in response to the mission complexity and the cognitive load (in particular MWL) of the human operator. This includes dynamic adaptation of the Automation Level (AL) and actual command/control interfaces, while maintaining stable MWL and the highest possible level of situational awareness of the human operator. Nonetheless, with physiological measurements the different methods are prone to various internal and external signal disturbances, which means that it is challenging to identify the true signal of interest from the noise. The comparison with other MWL measures, such as subjective questionnaires and objective task performance measures, are important for cross referencing with the physiological measures, in order to verify that they are correctly and accurately measuring MWL. In addition, the monitoring of multiple parameters in a sensor network is required, as well as data fusion methods, to ensure the accuracy and reliability of the MWL estimation. The additional measures are also promising for use as labels in Artificial Intelligence (AI) techniques such as supervised Machine Learning (ML). Although the measurement of the physiological response and inferring cognitive states (with and without system adaptation) was demonstrated in previous studies, there are still significant research gaps, one of which relates to a universally valid method for determining MWL that can be applied to any operational scenario. Henceforth, in this study we tested and analyzed physiological measures of MWL, including EEG and eye tracking, in a complex OTM UAS wildfire detection mission. Additionally, objective measures were explored, including a secondary task performance and controller inputs, in an analytical comparison with the physiological measures. Although subjective measures are the closest to a ground truth, at the moment they only provide a response at infrequent intervals during the mission and cannot capture the detailed MWL variations during the tasks without being disruptive. Lastly, a fusion approach with the physiological measures was performed and correlated with the task index. The results show that the correlation with the physiological measures and the task index were good for both physiological measures, with the strongest result when fusing the two measures. These results demonstrate the ability of measuring MWL in a complex UAS mission and will be used in further developments of the CHMS.

## Figures and Tables

**Table 1:** Primary and secondary mission objectives.

1. PRIMARY: Detect the Presence of Wildfire and Track the Spread of Detected Fires				
1.1 Wildfires are initialized at the start of the scenario, and spot fires may also be created during the mission. The fires propagate through the AOR based on environmental conditions.			1.2 While the UAVs are not equipped to fight the wildfires, authorities have requested for constant visual coverage to be maintained on the fire front (i.e., the segment of the map where a fire is still burning).	
**2. SECONDARY:**				
2.1 Maximize sensor coverage of the search area		2.2 Maintain a serviceable level of navigation and communication (comm) performance		2.3 Maintain a serviceable level of fuel
a. Sensor coverage over the AOR is tracked in terms of revisit time:	b. Three measures are used:	a. Navigation performance:	b. Communication performance:	a. UAVs with low fuel need to be sent back to base for refueling
i. Excellent revisit time means below 20 min ii. Adequate revisit time means between 20 to 30 min iii. Poor revisit time means more than 30 min. Implies that the area was not covered by the sensor at all.	i. Coverage of the active sensor (i.e., revisit time of the lidar) ii. Coverage of the passive sensor (i.e., revisit time of the IR camera) iii. Coverage of both sensors (i.e., revisit times of the lidar or IR camera)	i. Excellent navigation accuracy is below 10 m ii. Adequate navigation accuracy is between 10 and 25 m iii. Poor navigation accuracy is above 25m	i. Excellent comm strength is considered above 70% ii. Adequate comm strength is considered between 50% and 70% iii. Poor comm strength is considered below 50%	

**Table 2:** Task index calculation for each UAV.

Pending Secondary Tasks	Penalty
Poor navigation performance (accuracy above 25 m)	+1
Adequate navigation performance (accuracy between 10 and 25 m)	+0.5
Excellent navigation performance (accuracy below 10 m)	+0
Poor communication performance (comm strength below 50%)	+1
Adequate communication performance (comm strength between 50% and 70%)	+0.5
Excellent communication performance (comm above 70%)	+0
Critically low fuel (fuel needed to return to base less than 1.5× of fuel on board)	+1
Low fuel (fuel needed to return to base between 1.5× and 2× of fuel on board)	+0.5
Adequate fuel (fuel needed to return to base more than 2× of fuel on board)	+0
Autopilot mode in hold	+1
Autopilot mode off	+0
UAV not assigned into a team	+1
UAV is assigned into a team	+0
UAV does not have any sensors active	+1
UAV does have sensors active	+0

**Table 3:** All results from the ANOVA analysis.

	F Value	*p* Value
Subjective MWL rating	24.09	6.283 × 10^−5^
Subjective SA rating	25.82	4.497 × 10^−5^
Task index	88.47	6.563 × 10^−8^
Controller input	22.1	9.472 × 10^−5^
EEG index phase 1–5	16.44	4.115 × 10^−6^
EEG index phase 1–3	19.57	0.0002
Visual entropy	34.54	1.051 × 10^−5^

**Table 4:** Notable correlation coefficients for each participant (Task = task index, EEG = EEG index, V.ent. = visual entropy, Fused = weighted sum of EEG and V.ent.).

	Participant 1	Participant 2	Participant 3	Participant 4	Participant 5
Task-EEG	0.354	0.662	0.567	0.677	0.878
Task-V.ent.	0.441	0.799	0.761	0.606	0.631
Task-Fused	0.479	0.845	0.757	0.724	0.824
EEG-V.ent.	0.411	0.578	0.572	0.570	0.673

**Table 5:** All feature comparison. Mean and respective standard deviation across all subjects.

	EEG Index	Visual Entropy	Task Index	Control Input	Fused Phys.	Fused Other
EEG index	1	0.561 ± 0.09	0.628 ± 0.19	0.037 ± 0.34	N/A	0.464 ± 0.24
Visual entropy		1	0.648 ± 0.14	0.167 ± 0.42	N/A	0.546 ± 0.13
Task index			1	0.205 ± 0.38	0.726 ± 0.14	N/A
Control input				1	0.135 ± 0.39	N/A
Fused phys.					1	0.541 ± 0.21
Fused other						1
